# Glucose Metabolism and Dynamics of Facilitative Glucose Transporters (GLUTs) under the Influence of Heat Stress in Dairy Cattle

**DOI:** 10.3390/metabo10080312

**Published:** 2020-07-31

**Authors:** Zaheer Abbas, Abdul Sammad, Lirong Hu, Hao Fang, Qing Xu, Yachun Wang

**Affiliations:** 1 Institute of Life Sciences and Bio-Engineering, Beijing Jiaotong University, Beijing 100044, China; zaheerabbas@bjtu.edu.cn (Z.A.); 18121612@bjtu.edu.cn (H.F.); 2 National Engineering Laboratory for Animal Breeding, Key Laboratory of Animal Genetics, Breeding and Reproduction, CAST, China Agricultural University, Beijing 100193, China; drabdulsammad1742@yahoo.com (A.S.); B20193040324@cau.edu.cn (L.H.)

**Keywords:** heat stress, dairy cattle, glucose, lactose, energetic metabolism, facilitative glucose transporters (GLUTs)

## Abstract

Heat stress is one of the main threats to dairy cow production; in order to resist heat stress, the animal exhibits a variety of physiological and hormonal responses driven by complex molecular mechanisms. Heat-stressed cows have high insulin activity, decreased non-esterified fatty acids, and increased glucose disposal. Glucose, as one of the important biochemical components of the energetic metabolism, is affected at multiple levels by the reciprocal changes in hormonal secretion and adipose metabolism under the influence of heat stress in dairy cattle. Therefore, alterations in glucose metabolism have negative consequences for the animal’s health, production, and reproduction under heat stress. Lactose is a major sugar of milk which is affected by the reshuffle of the whole-body energetic metabolism during heat stress, contributing towards milk production losses. Glucose homeostasis is maintained in the body by one of the glucose transporters’ family called facilitative glucose transporters (GLUTs encoded by *SLC2A* genes). Besides the glucose level, the GLUTs expression level is also significantly changed under the influence of heat stress. This review aims to describe the effect of heat stress on systemic glucose metabolism, facilitative glucose transporters, and its consequences on health and milk production.

## 1. Introduction

Glucose is a universal fuel for the energy metabolism and biological synthesis pathways of all animal cell types [1]. In dairy cattle, dietary carbohydrates provide a major source of energy for maintenance, growth, and production. Animal cells require glucose for oxidative and non-oxidative, adenosine triphosphate (ATP) production, and for anabolic reactions that produce various vital sugars [2]. Several cell types and tissues such as the brain, red blood cells, kidney, and mammary tissues have the utmost need for glucose as a substrate [3]. Therefore, glucose is an essential fuel for all the organisms and it needs to be permanently available at an adequate level in the blood. Glucose is the main precursor for lactose synthesis and is therefore required in large amounts by lactating dairy cows to cover the milk demand. Milk yield largely depends on the synthesis of mammary lactose because of its osmoregulatory function for mammary water uptake. Therefore, the glucose supply to the mammary tissues is a critical regulator for milk synthesis [4]. Because of its hydrophilic nature, glucose cannot penetrate the lipid bi-layer, hence, to facilitate the diffusion of glucose along the concentration gradient, specific carrier proteins are required [5]. Thus, glucose is absorbed by the mammary epithelial cells through a passive process, encouraged by the downward glucose concentration gradient through the plasma membrane [6].

Glucose uptake is arbitrated by the family of facilitative glucose transporters (GLUTs) and sodium-glucose co-transporters (SGLTs). This facilitative glucose transporters family is further divided into three classes. Class I (e.g., GLUT1) is responsible for basal levels of glucose uptake and is present in all types of cells. Class II, such as GLUT10, has a high chemical attraction for glucose, while class III (e.g., GLUT5) is responsible for fructose uptake [7]. The intestinal uptake of glucose is mainly in two steps: firstly, the absorption of glucose and galactose is done by the apical brush border which is carried out by the sodium-dependent glucose co-transporter1 (SGLT1) while uptake of fructose is mediated by the GLUT5, secondly, diffusion of glucose, fructose and galactose from the intestine to the blood capillaries is facilitated by the GLUT2 and, in humans, also by GLUT5 [8].

Climate change threatens the survivability of animal species, ecosystems, and the keeping up of livestock production systems around the world, especially in countries with subtropical climatic conditions [9]. Heat stress provokes acclamatory responses which are essential in the preservation of cell survival. Physiological responses to heat stress are reduced feed intake, elevated rectal temperature, respiration rate, and heart rate [10]. Hormonal changes like thyroxin, tri-iodothyronine, cortisol, and insulin are particularly accountable for the change toward glucose and energetic metabolism [11]. Biochemical change includes changes in various antioxidants, enzymes, and metabolites like blood glucose level, while hematological response includes changes in hematocrit value, hemoglobin concentration, and erythrocyte number [12]. Molecular changes in the gene expression of heat shock proteins [13] and reshuffles in amino acid concentration and skeletal muscle metabolism bears major consequences for the heat stress effect on dairy cattle [11]. All these physiological, biochemical, and molecular responses make the animal survive in a harsh and stressful environment [14]. The study performed in mice showed that heart glucose level changes as a response to heat stress within a different period (12 and 24 h) of exposure to heat stress [15]. Besides, the study performed in chicken revealed that the apical glucose absorption in the jejunum is elevated to compensate for the glucose level in blood during heat stress [16]. Different breeds of dairy cows experience significant changes in blood chemistry besides milk production, rectal temperature, and respiratory rate [17]. Various studies performed regarding the effect of heat stress on glucose level in the last decade show a significant decrease in blood glucose in dairy cattle [18,19,20]. Moreover, dairy goat also shows a significant decrease in the whole-body turnover of glucose under heat stress [21]. This alteration in glucose is due to the acclamatory adaptations to heat stress, which include reduced feed intake along with a metabolic reshuffle governed by hormonal changes. During heat stress the insulin level increases that suppresses the glucose level and non-esterified fatty acids (NEFA) by its antilipolytic activity, thus disturbing the energetic state of the cow and ultimately affecting the milk production. Moreover, the glucose level is also governed by the tissue-specific glucose transporters like GLUTs. Since GLUTs are responsible for the glucose absorption, the distribution of glucose has been significantly affected in various studies by heat stress [22,23]. Considering the metabolism of glucose and the role of GLUTs in glucose transportation under heat stress, the importance and role of GLUTs in coping with heat stress is of immense importance. Therefore, this review aims to give an understanding of the systemic glucose metabolism under normal and heat-stressed conditions as well as the role of facilitative glucose transporters in these procedures in dairy cattle. This review also gives up-to-date information about the structural, functional, and molecular characterization of the glucose transporters in cattle and its possible role in heat tolerance.

## 2. Physiology of Glucose Metabolism

**Figure 1:**
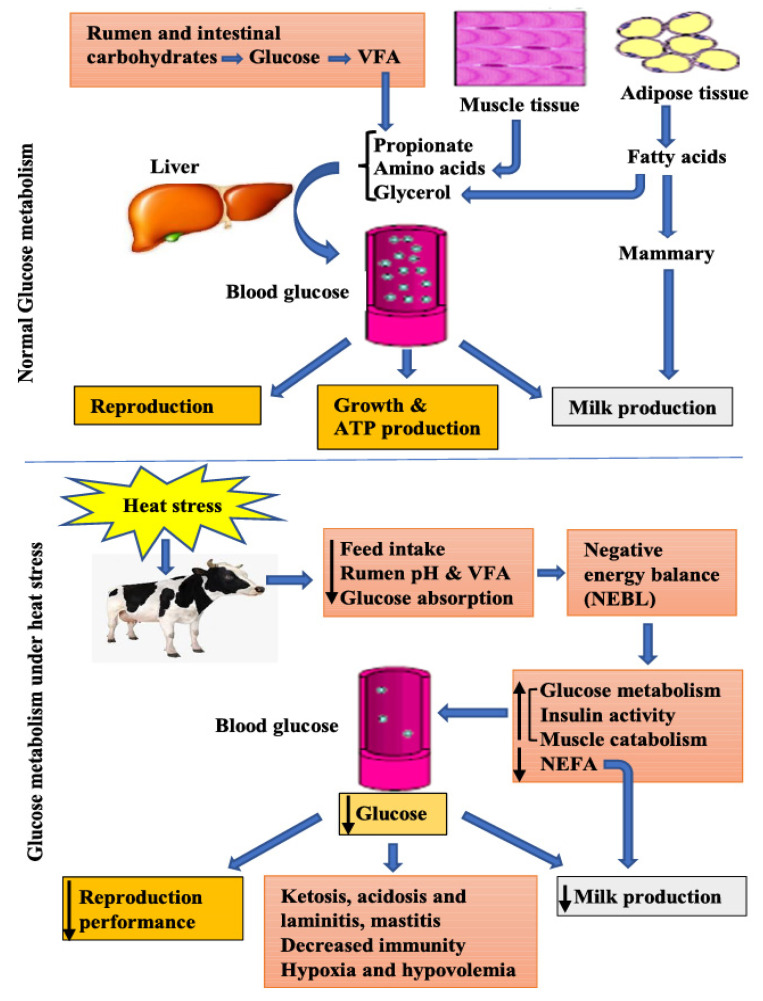
Glucose metabolism under normal and heat-stressed conditions in cattle.

Tissues depends on energy substrates like carbohydrates, which are carried within the plasma to be absorbed by various tissues and organs according to their requirements. In contrast to other nutrients, the product of lipolysis, NEFA, and tri-acylglycerols, are sustained within tight limits in dairy animals. Understanding glucose synthesis and metabolism, nutritional glucose accessibility, and the process of gluconeogenesis in the maintenance of glucose homeostasis is of utmost importance for the operation of the production and quality of agricultural foods [24]. Glucose is mainly synthesized from feed, from hepatic glucogenesis, or mobilization of glycogen warehoused inside the body [25]. Ruminal microorganisms are gifted for the digestion of fibrous feed that enables them to eat and partially digest plant cellulose and hemicellulose, which results in the formation of fatty acids, propionate, acetate, and butyrate [2]. Thus, glucose is then re-synthesized in the liver from these volatile fatty acids (VFAs) as well as amino acids and glycerol by using a process called gluconeogenesis (Figure 1). Hence, gluconeogenesis is extremely important to ruminants because it provides 75% and 90% of the total glucose needs in neonatal and adult ruminants, respectively [26].

## 3. Regulation of Lactose (Milk Glucose) in Dairy Cattle

Glucose supply in the lactating dairy cow is very essential due to its demands for milk synthesis. The mammary gland lacks glucose-6-phosphatase enzyme, therefore it cannot synthesize glucose by itself from other precursors [27]. Thus, the mammary tissue relies on the blood glucose supply for milk synthesis; 72 g of glucose is needed to produce one kilogram of milk [28]. During the transition period, the high-yielding dairy cows are put to the challenge of acquiring more energy for increased milk production as well as maintenance [29]. High-energy metabolism activities commence after parturition in the portal-drained viscera (PDV), which together with high liver glucose, meet the lactation demands [30]. During this period of higher mammary activity, liver energy disposal is higher than PDV output, reflecting higher energy consumption by non-PDV organs. However, as lactation proceeds, splanchnic flux became more positive (high-energy disposal of liver subsidies), probably representing the return of feed-derived lactate being diverted towards body energy reserves as fat [31]. Splanchnic circulation is the blood flow from celiac and mesenteric arteries towards abdominal organs. It receives about 25% of the heart blood output and thus maintains a constant percentage of the blood volume under normal conditions [32]. The increase in demands for energy can be partially fulfilled by increased feed utilization, but is limited because of low dry matter intake and a decrease in appetite during the transition period as well as during heat stress, and thus tends to stimulate mobilization of body reserves [11,31]. In the high-milk-yielding cows, the mammary glucose uptake accounts for nearly all of the glucose supply, proposing a smaller amount of glucose for other body tissues [33]. Beside lactose synthesis, glucose also has a prominent effect on the mammary cell viability and proliferation that shows a very close relationship between blood glucose and lactose regulation [34].

## 4. Heat Stress Effect on Glucose Metabolism

When the core body temperature of animals exceeds the range specified for normal activities, it provokes the acclamatory response to neutralize the effect of heat stress, resulting in significant changes in the blood metabolites and glucose level [35,36]. Although changes in the body glucose level depend on the intensity and the duration of heat stress, acute heat stress did not affect the glucose concentration in broiler and rats [37,38], while chronic heat stress decreased circulating glucose levels in bulls [39], cows [40], and broilers [37]. Therefore, it is evident that the change in the body glucose level depends on the intensity and the duration of heat stress. In dairy cattle, alterations in body glucose during heat stress are due to the reduced feed intake and energy (carbohydrates) balance, pushing the cow to enter an energy de-stabilized condition, and this is independent of the lactation stage, essentially due to reduced feed intake and energetic metabolism modulation [41,42]. 

Despite reduced feed intake, the heat-stressed animal shows post-absorptive changes in glucose level that are independent of the energetic status of the animal [11,41]. The alteration in postabsorptive carbohydrate metabolism is due to the increase in basal- and glucose-stimulated insulin levels [19,43]. The reasons for increased insulin level are not yet clearly understood, but they are probably protective and adaptive in nature [41]. These include the activation and up-regulation of HSPs, hyperprolactinemia, high intracellular concentration of Ca^+^, immune response toward endotoxin (LPS), and oxidative stress [44,45]. Glucose regulation is not only governed by the insulin, as there are several insulin-independent glucose transporters (GLUTs) that show distinct affinities for glucose, e.g., heat stress upregulated in vitro GLUT1 [46].

### 4.1. Decreased Feed Intake and Negative Energy Balance

Heat stress has a direct adverse effect on the appetite center of the brain, reducing feed intake. To reduce internal heat production, animal reduce feed intake, and thus less heat needs to be dissipated [41]. In high-yielding cows, high feed intakes and milk production make them more susceptible to heat stress than the low-yielding cows. To compensate for the reduction in feed intake and heat stress, the animal uses the internal body reservoirs to support the maintenance and production [47]. Moreover, the maintenance requirements of the animal increase by 30% in heat-stressed dairy animals [9]. The reduction in feed intake, increased maintenance cost, and sustaining milk production lead heat-stressed cows to experience negative energy balance (NEBAL), and thus jeopardize the animal’s production, reproduction, and health [41,48]. In terms of production losses, reduced feed intake is responsible for decreases of milk of up to 35% in lactating cows [18], while another study showed a 50% decrease in milk production due to the reduced feed intake during heat stress. This decrease in milk production is due to the feed-intake-dependent and -independent post-absorptive changes in glucose uptake due to heat stress [19]. Furthermore, oxidative stress and hypoxia due to the blood flow redistribution toward the periphery during heat stress cause leaky gut, and thus the entry of LPS in the blood through intestinal barrier impairment causes endotoxemia. This triggers the immune response, which needs more energy in the form of glucose, and thus glucose sparing for milk production is affected.

### 4.2. Heat Stress Effect on Ruminal and Intestinal Glucose Processing

In ruminants, the main source of carbohydrate is the feed containing cellulose, hemicellulose obtained from the fibrous feed, and starch from the grains [49], which is disturbed due to the low feed intake under heat stress to avoid heat production by digestion and metabolism [50]. During heat stress, a large amount of blood flows toward the periphery to dissipate heat, thus decreasing gastrointestinal uptake of glucose, and also the VFAs accumulate in the rumen, which lowers the pH. The amount of saliva that is normally deposited to the rumen decreases because of increased droll, which thus decreases rumen pH, and compromises rumen health by increasing rumen acidity [51]. Disturbances in rumen health due to heat stress significantly decrease total VFAs, acetic acids, and propionic acid formation, which are the main contributors to glucose production [52]. In short, the rumen inappropriate function due to the heat stress is a main contributor to the decrease in whole-body glucose.

### 4.3. Liver Metabolism of Glucose under Heat Stress

The liver plays an important role in glucose homeostasis by producing glucose from ruminal propionate, muscle tissue amino acids, and adipose tissue contribution of glycerol using a process called gluconeogenesis under normal conditions [25]. As mentioned earlier, heat stress encourages a reduction in feed intake that lowers the whole-body glucose, thus enhancing the anaerobic glycolysis in the liver by upregulating the lactate and pyruvate concentration to overcome the NEBAL situation produced by reduced feed intake under heat stress [53]. In the same study, the level of amino acids is decreased in the heat stress group, indicating the increased conversion of these metabolites into glucose to stabilize glucose homeostasis and energy supply during NEBAL in dairy cows [18]. However, this increase in liver glucose synthesis may not be enough to compensate for the whole-body reduction in glucose during heat stress [11]. Decreased growth hormone (Somatotropins) and IGF-II are typical to heat stress and alter the feed intake of cows [54]. Similarly, the GH receptor abundance is reduced in the liver, which, combined with low *IGF-I* mRNA abundance, defines the alterations in feed intake and gluconeogenesis [18,54], liver and mammary tissues’ contribution to milk yield decline, and acquiring acclamatory homeostasis, under heat stress [55].

### 4.4. Adipose Tissues Contribution to Glucose Metabolism under Heat Stress

Adipose plays an important role in the production of glucose by the liver during the glucose-deficient state; NEFA acts as a metabolic substrate to regulate glucose that is indirectly governed by the insulin [56]. Normally, during NEBAL, because of the transition period or malnourished state NEFA are exported from adipose tissue by sending the lipolytic response to β-adrenergic that inhibit blood insulin and induce systemic insulin, which triggers lipolysis and inhibit glucose utilization [57]. However, during heat stress this phenomenon is completely opposite because the basal plasma NEFA concentration is reduced in cattle. Insulin shows a potent antilipolytic activity, and that is why heat-stressed animals lack the mobilization of adipose and triglycerides [11,58]. Heat stress induces the lipo-protein lipase instead of mobilizing NEFA, suggesting triglycerides’ anabolism. Limited adipose tissue utilization during heat stress suggests the prevention to employ the glucose sparing mechanism for the milk production and skeletal muscle activities [59]. This avoiding of adipose mobilization and increased glucose expenditure is probably a strategy to reduce heat production due to metabolic activities [41]. The deficiency of NEFA for oxidative purposes is paired with the decrease in VFAs’ availability, leaving glucose and amino acids as the available sources of oxidative substrates. Thus, glucose is utilized as the main oxidative fuel in heat-stressed animals [59]. From the above discussion, it is clear that the adipose metabolism is characterized by low NEFA response due to high insulin activity, exuberating the existing NEBAL, thereby jeopardizing the health, welfare, and production of heat-stressed cows.

### 4.5. Nexus of Protein and Glucose Metabolism under Heat Stress

Skeletal muscle is the main source of amino acids as well as stored glycogen that is recycled by the liver for the glucose supply to support lactation under thermo-neutral conditions. As the muscle has a reduced ability to oxidize fatty acids, it presumably depends on circulating and stored glucose for its energy requirements. Evidence suggested that the protein catabolism elevates as a result of increased use of body fat during heat stress which alleviates the glucose deficiency to some extent, as the glycogenic amino acids provide energy through the tri-carboxylic acid (TCA) cycle or by gluconeogenesis [60]. The regulation of pyruvate entry to the TCA cycle plays a major role to favor lactate and pyruvate-alanine flux to hepatic gluconeogenesis [60,61]. Heat stress exerts a major effect on the amino acid metabolism, resulting in an increased mobilization of skeletal muscle protein [19]. During heat stress reduction in milk, changes in protein mobilization suggest that more amino acids are required for the glucose production through the liver to support immune function and energy needs rather than milk production [36]. Therefore, during heat stress, the muscle protein catabolism is increased, which seems to fulfill the amino acid availability for the hepatic glucose production instead of the direct oxidation [62]. Metabonomic investigation revealed that heat stress lowers the blood glucose, but increases the pyruvate and lactate along with the activity of the lactate dehydrogenase [63]. High insulin activity and amino acid catabolism in gluconeogenesis causes changes in blood amino acids [64], while, at the same time, a decrease in milk protein content was observed during heat stress [36]. High variations in the hemoglobulin, packed cell volume, glucose, total protein, and albumin level have been observed in the plasma of thermal exposure individuals [65]. Muscle anabolism and nucleic acid synthesis biomarkers are affected [66]; markers of protein catabolism such as plasma nitrogen, 3-methyl-histidine, and creatine increases [67], while blood lysine levels are also shown to decrease under heat stress. Milk composition analysis of heat stress cow shows the alterations to alpha and beta-casein levels [36]. It is clear that muscle catabolism supports gluconeogenesis [62], therefore muscle catabolism during heat stress leads to the deterioration of milk quality and quantity [66].

## 5. Lactose Regulation under Heat Stress

Dairy animals require glucose to form milk lactose, thus lactose production is the primary osmo-regulator as well as a determinant of milk yield [51]. Mammary utilization of glucose and long-chain fatty acids are suggested to be the main contributor to milk volume and mammary efficiency respectively [28]. To generate less metabolic heat during heat stress conditions, the skeletal muscle still appears to consume glucose at an increasing rate. As a result, the mammary gland does not utilize enough glucose which results in decreased mammary lactose production as well as milk yield. This may be the primary mechanism and is responsible for the extra reduction in milk yield that cannot be explained by reduced feed intake [51]. However, milk glucose (lactose) is significantly affected by the heat stress and the obtained values for the percentage of lactose varied (4.45 ± 0.54% in spring versus 4.03 ± 0.24% in the summer period in dairy cattle [68]. Different studies performed in dairy cattle revealed that heat stress significantly decreases the blood glucose level as well as lactose (milk glucose) that is accountable for the decreased milk production (Table 1). Whole-body turnover of blood glucose in dairy goats was significantly down-regulated as a result of both moderate and severe heat exposure [21]. Under the influence of heat stress, blood glucose declines despite the increase in the intestinal glucose absorptive capacity [16], the elevation of the renal glucose re-absorptive capacity [69], and the increased liver glucose output [70]. The increased glucose pool entry combined with the low blood glucose might suggest an increased rate of glucose parting the circulating blood pool. Thus, glucose becomes the preferred fuel of heat-stressed animals.

## 6. Facilitative Glucose Transporters (GLUTs)

The transportation of glucose through the plasma membrane is governed by the two well-known processes named as passive—that is, an energy-independent process regulated by the facilitated glucose transporters family (GLUTS) encoded by the *SLC2A* genes. The second type of transportation is active and energy-dependent coursed by the sodium-dependent glucose transporters family (SGLTs) encoded by the *SLC5A* genes [72].

**Figure 2:**
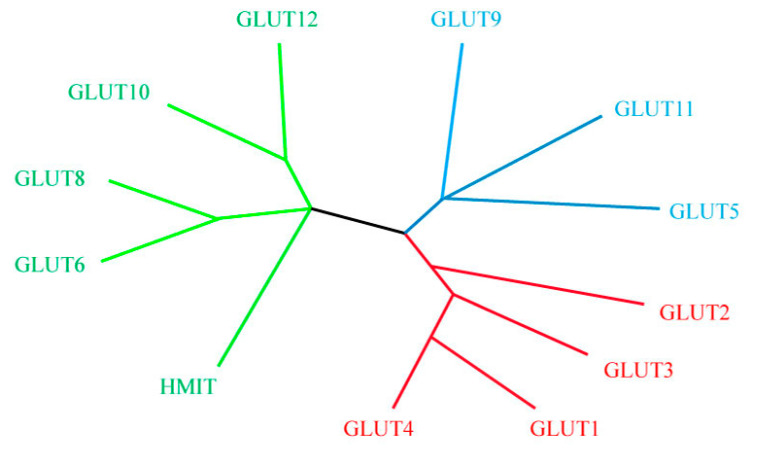
Un-rooted phylogenetic tree of the 12 bovine family members of the Facilitative glucose transporters (GLUTs)1 (Małgorzata et al., 2005) [73].

Furthermore, the GLUTs family is comprised of three classes: Class I includes GLUT1, GLUT4, GLUT3, and GLUT2, that are 65, 66, and 54% matching in bovine, respectively. Class II includes GLUT9, GLUT11, and GLUT5 (fructose transporters), and are 56% and 43% alike, respectively. Class III contains GLUT6, GLUT8, GLUT10, GLUT12, and H^+^-myo-inositol co-transporter (HMIT), with a similarity of 43, 63, 26, and 29% in their amino acid sequence, respectively, as shown in the Figure 2 [73].

**Figure 3:**
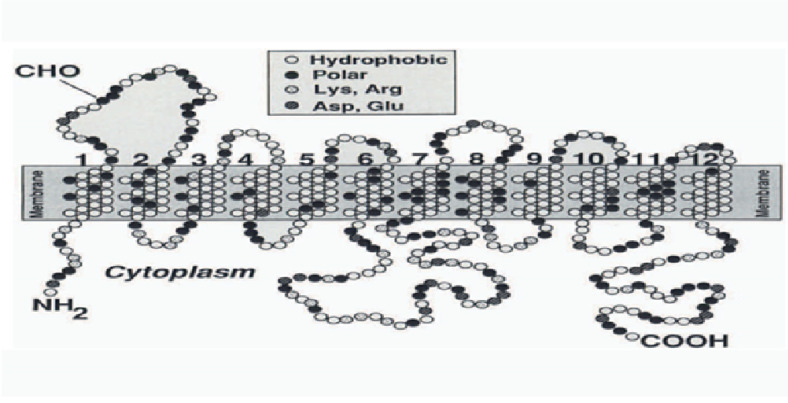
Schematic membrane topology of facilitative glucose transporters ((Małgorzata et al., 2005) [73].

The GLUTs family (solute carriers; gene symbol *SLC2A*) comprises proteins that facilitate the absorption of glucose (and other sugars) through the plasma membrane in an energy-independent process [74]. Each GLUTs protein includes 12 trans-membrane-spanning domains with intracellular carboxy and amino termini, as shown in Figure 3. Inhibitors, e.g., phloretin, phlorizin, and cytochalasin B, can efficiently block the diffusion of glucose by these proteins [75].

These proteins are expressed in certain ways in tissues and cells that exhibit unique dynamics and regulatory characteristics, reflecting their specific functional roles [72]. Each GLUTs plays a definite role in metabolic activities, depending on its tissue- and substrate-specific expression in various biological conditions [8]. The additional genomic information along with the tissue localization and functional characteristics of the GLUTs family are summarized in Table 2. 

## 7. Heat Stress Effect on Facilitative Glucose Transporters (GLUTs) Family

The increase in ambient temperature provokes kind of adaptations, like alterations in protein synthesis, which may be due to plasma amino acid concentration and a lower energy supply. [87]. This compensation to energy level activates many proteins, including glucose transporters as well. In chickens, heat stress has shown a pronounced effect on the expression of GLUTs family proteins, e.g., GLUT1 was significantly downregulated in the ileum, while the expression of GLUT5 and GLUT10 was upregulated in chicken ileum in the heat-stressed group compared to the thermoneutral group (Table 3) [22]. The expression of GLUT1 is reciprocal to the circulating glucose level within the body [88], also, during heat stress, the plasma glucose level increases, which tends to decrease the GLUT1 expression [89]. The facilitative glucose transporter like GLUT5 is accountable for the absorption of glucose and fructose from the intestine, while GLUT10 is responsible for the uptake of glucose, dehydroascorbic acid, and galactose in the brain, lungs, heart, kidney, pancreas and skeletal muscles. GLUT10 increases the supply of dehydroascorbic acid to the mitochondria, causing a reduction in reactive oxygen species (ROS) level during heat stress; the increase in expression is to increase the supply of ascorbic acid [90]. Another study performed in broiler chicken showed that heat stress down-regulates the expression of GLUT2 glucose transporter [89]. However, in contrast to chicken, heat stress increases the intestinal expression of GLUT2 protein in pigs [91]. The expression of GLUT4 under heat stress was studied in many tissues including the liver and muscle of growing pigs that favored higher expression of GLUT4 under heat stress [92]. The glucose transporter SLC2A3 (GLUT3) gene has been studied in cultured Sertoli cells of boar under heat stress that show increased mRNA expression of the *SLC2A3* gene [93]. A comparison study of the expression of HSP70 and GLUT1 in Indian buffaloes showed a significant increase in GLUT1 protein under heat stress [23].

The energy compensatory mechanisms under the influence of heat stress are homeostatic in the early phase, characterized by physiological modifications that are themselves energy-intensive and requires glucose combustion in enormous quantities [94,95]. However, increasing the influence of heat stress debilitates the dissipation capability of the dairy cows, and a series of acclamatory responses commence which are homeorhetic in nature. These homeorhetic responses are truly endocrine and molecular in nature [10], as also indicated in the aforementioned discussion of various species, where glucose transporters are modulated at various levels under the influence of heat stress. Here, we want to mention that the digestive system can be disturbed by the heat stress [96,97], which has consequences for energetic metabolism and absorption [98], physiological integrity [99], and possible immune system repercussions [100]. All these alterations can constitute additional energy transport [91], availability [101], and expenditure [44], and underlying this modulation of energy dynamics is very important in this context under the influence of heat stress. The field of molecular study pertaining to the glucose transporters has largely been neglected among dairy cattle, and indeed many studies have mentioned this as a potential venue of future research towards the understanding of energetic metabolism under the influence of the ever-increasing constraint of heat stress regarding dairy production [11,41].

## 8. Polymorphism in Facilitative Glucose Transporters (GLUTs) Bringing Sustainable Improvements in Energy Dynamics in Dairy Cattle

The identification of genetic markers and quantitative trait loci related to improvements in energy status and production under heat stress will surely form the basis of genomic selection for thermal tolerance [11]. The identification of such valuable markers requires certain appropriate phenotypes so that accurate genetic effects can be predicted [102]. In the above discussion, we referenced some important studies which have predicted the differential level of expression of certain metabolites, amino acids, and hormones [36,53,103] that can be regularly recorded as phenotypes for the individual energetic metabolic status among dairy cows. Milk fats and protein level and composition are the additional parameters indicative of heat stress and energetic metabolism, and could be focused on as measurable phenotypes. Besides convenient milking line measurements, milk temperature measurement as the reliable substitute for the individual dairy cow body temperature offers additional benefits in this regard [104]. Combining physiological manifestations of dairy cattle under heat stress, novel relevant phenotype measurements [105], and the integration of molecular techniques [102] could make the journey towards thermo-tolerance more achievable. 

The genetic polymorphism in *SLC2A* family genes can alter the gene expression or functions of glucose transporter proteins. Thus, they may have consequences for the energy homeostasis of the animal, and glucose supply alterations towards the mammary gland can affect milk yield. Additionally, DNA sequence variations in the GLUT1 encoded gene in humans have been identified as related to the development of certain disease like diabetes or cancer [106,107]. A lot of next-generation studies have accumulated hundreds of SNPs in genetic databases related to GLUTs family encoding genes. The drawback is that these SNPs have not been validated with other methods and their association with production or functional traits remains to be elucidated. A study found six SNPs in exons and intronic regions, which were significantly associated with milk traits in dairy cattle. [108]. The molecular dynamics of glucose transporters and their complex interactions with the endocrine system at different levels and biological conditions are a way forward in the understanding of this subject in dairy cattle. Discovering functional polymorphisms in the bovine facilitative glucose transporter genes, strong genetic effects related to the level of glucose and other markers of energetic metabolites combined with relevant phenotypes, constitute future avenue in this context.

## 9. Mitigation Strategies towards Heat Stress and Its Consequences

Heat stress is a major factor that can negatively affect milk production [109], as well as nutrient supply for the reproduction and to maintain the health of the cow [48]. In this scenario, mitigation approaches are required to reduce the severity of the heat stress effects on dairy production. Short-term mitigation strategies include improved nutrition and proper herd management. While long-term strategies are achieving adaptability towards heat stress and development of thermo-tolerance through selective breeding [11].

Dairy cows’ response towards heat stress is variable based upon the differences in breed, production, location, housing, and husbandry practices, to name a few [110,111,112]. In this context, the actual prediction of heat stress load on cattle is of paramount importance [113]. Temperature-Humidity Index (THI) is a widely accepted criterion of heat stress measurement, while generally cows experience heat stress at 25 °C. However, recently researchers are focusing THI calculation at the most ambient level, considering the actual micro-climates around cows. This approach of THI evaluation, combined with the physiological parameters of heat stress, is essential to depict the actual heat stress magnitude of cows [114]. By this way, managers can monitor heat stress events and design appropriate housing and husbandry to attain heat stress abatement through appropriate cooling, management, and nutrition support aids [110]. Additionally, the aforementioned proper heat stress assessment strategies would also help to identify the thermo-tolerant individual cows. 

As increased milk production is positively correlated to both dry matter intake and subsequent metabolic heat production [112], nutrition support decisions are important. In the presence of many alterations to the digestive physiology and profound NEBAL, the importance of nutritional strategies of heat stress abatement is two-fold [11]. The adverse effects of heat stress stemming from reduced feed intake and alterations in gastrointestinal can be manipulated by the interventions in cattle nutrition [50]. Feed supplements like multi-vitamins, minerals, and essential amino acids are recognized as helpful in maintaining rumen function, improve the immune system [115] and milk production during heat stress [116,117,118]. It is well documented that heat stress reduces feed intake which negatively influencing nutrient absorption and affecting the immune system as well as the immune response [119]. Energy-rich diets [120], dietary fats [121,122], and rumen degradable proteins [123], are important primary feeding stuffs to be cared for, while dietary yeasts [124], fermentates, betaine [125], and Dietary Cation–Anion Difference [126] feeding would augment the constrained gastrointestinal tract and the metabolic status of dairy cows under heat stress. Among the many other adverse effects of heat stress and energy-rich diets is ruminal acidosis, which could be overcome by feeding bicarbonates [11]. Amino acids like propionate supplementation have been shown to improve energy metabolism status and milk yield as it is the primary source of glucose production [127]. Multivitamins like vit-B complex, vit-C, vit-E, and Niacin and Nicotinic acid improve the immune system as well as being helpful in maintaining general health during heat stress [128]. Minerals supplementation such as Mn, Zn, Mo, P, and Se are proven to improve metabolic status as well as the health of the dairy cows [118]. Ionophores and Monensin have shown a positive effect on production parameters as well as energetic metabolism during heat stress [20,129].

In order to secure dairy production under the influence of ever-increasing events of heat stress, sustainable long-term mitigation strategies include achieving thermo-tolerance among dairy cows. Modern data collection technologies of heat stress and activity monitoring give a huge amount of data that could be used for modeling to identify thermo-tolerant cows [105,130,131]. Crossbreeding with thermo-tolerant animals, preferably within the breed, is a way forward in this context [132,133]. The basic idea of thermo-tolerance breeding includes identification of thermally adapted cows [105], with due consideration to the relevant phenotype [102], and thus, having thermo-tolerant cows with good adaptability towards the challenge of heat stress conditions [94]. The detection of genetic markers associated with improved energy metabolism and production would provide the basis for the thermo-tolerance capability of dairy cattle [11] and help in understanding the biological mechanisms of thermo-tolerance [102], such as differential expression of certain genes, metabolites, amino acids, and hormones [53,61]. This obtained information can thereby be used for the identification of genes and genomic regions along with associated phenotype measurements responsible for thermo-tolerance, that can be ultimately used in genomic selection [102]. In short, regarding the physiological response of cattle under heat stress, the identification of novel related phenotypes [105], and integration of molecular technique [102] can make permanent, cumulative, and cost-effective breeding for thermo-tolerance possible.

## 10. Conclusions

Glucose is a major fuel for the body functions as well as milk production and is regulated according to the metabolic changes governed by high insulin level and tissue-specific glucose transporters GLUTs during heat stress. Glucose transport dynamics, reciprocal relationships, and modulation under heat stress, together with relevant phenotypes assessment, need to be explored in detail. Few studies have been carried out in terms of heat stress effect on glucose and glucose transporters in dairy cattle and the discovery of relevant biomarkers. Therefore, this review gave an understanding of the previous relevant literature and the use of those tools and approaches to carry out a systemic study of glucose level, GLUTs and heat stress nexus. In order to further the progress towards sustainable dairy production under heat stress, the application of proper mitigation strategies is of the utmost importance. Heat stress assessment, adequate cooling measures, and nutrition support decisions with additional feed supplements are the short-term mitigation approaches. Moreover, the identification of genetic markers associated to energetic metabolism and production could lead to long-term adaptation to heat stress. GLUTs have a certain role in this context and the encoding *SLC2A* family of genes has a moderate variability reported, and opens a promising avenue to confer improved energetic status and contribute to thermo-tolerance in dairy cattle.

## Figures and Tables

**Table 1:** The effect of heat stress on the blood glucose, milk lactose, and milk yield in dairy animals.

Specie	Glucose Level HS/TNZ	Milk Lactose HS/TNZ	Milk Yield HS/TNZ	*p*-Value	Reference
Cow	6.3 mg/L ↓	0.12% ↓	6.65 kg ↓	*p* < 0.05	[19]
Cow	3.15 mg/L ↓	0.06% ↓	5.25 kg ↓	*p* < 0.05	[20]
Cow	6.5 mg/dL ↓	0.14% ↓	7.5 kg ↓	*p* < 0.05	[18]
Cow	------	0.42% ↓	3.14 kg ↓	*p* < 0.05	[68]
Goat	202 µmol ↓	11% ↓	11–13% ↓ (0.21 kg)	*p* < 0.05	[71]

HS (heat-stressed), TNZ (thermo-neutral zone), mg/dL (milligram per deciliter), µmol (Micro molar).

**Table 2:** Genomic information, tissue localization, and functional characteristics of facilitative glucose transporter (GLUTs) family members.

Protein	Gene	Chr. Location	Exon No.	Accession No		Protein Size	Main Tissue Localization	Functional Characteristics	References
				Gene	Protein				
GLUT1	*SLC2A1*	Chr.3	10	NC_037330.1	NP_777027.1	492 aa	Mammary gland, kidney, brain, omental fat, skeletal muscle, bovine follicle, bovine ovary, and corpus luteum	Basal glucose transport across blood tissue barriers	[76,77]
GLUT2	*SLC2A2*	Chr.1	11	NC_037328.1	NP_001096692	510 aa	Small intestine, liver, Islets, kidney, and jejunal region	Glucose (low affinity)	[78,79]
GLUT3	*SLC2A3*	Chr.5	11	NC_037332.1	NP_777028	494 aa	Bovine ovary, follicles, corpus luteum, and brain.	Glucose (high affinity	[80]
GLUT4	*SLC2A4*	Chr.19	11	NC_037346.1	NP_777029	509 aa	Heart, muscle, brain and adipose tissue	Transport of glucose in all insulin-responsive tissues	[78]
GLUT5	*SLC2A5*	Chr.14	13	NC_037341.1	NP_001094512	501 aa	Small intestine, testes, kidney, muscle, brain and adipose tissue	Fructose (high affinity), glucose (low affinity)	[79,80,81]
GLUT6	*SLC2A6*	Chr.11	10	NC_037338.1	NP_001073725	507 aa	Brain, spleen, and peripheral leukocytes.	not determined	
GLUT8	*SLC2A8*	Chr.11	10	NC_037338.1	NP_963286	478 aa	Mammary gland, testis, kidney, intestinal epithelia, skeletal muscle, blastocyst and liver	Insulin-responsive transport in blastocyst	[82]
GLUT9	*SLC2A9*	Chr.6	18	NC_037333.1	XP_002688502	506 aa	Kidney and liver	not determined	[5]
GLUT 10	*SLC2A 10*	Chr.13	5	NC_037340.1	NP_001179368	536 aa	Liver and pancreas	not determined	[83]
GLUT 11	*SLC2A 11*	Chr.17	12	NC_037344.1	NP_001180026	496 aa	Heart, muscle (short form) liver, lung, trachea, and brain (long form).	Glucose (low affinity), transport of fructose (long form)	[84]
GLUT 12	*SLC2A 12*	Chr.9	7	NC_037336.1	NP_001011683	621 aa	Skeletal muscle, spleen, kidney, testes, mammary gland, liver, lung, and intestine	Insulin-dependent glucose uptake in mammary gland	[85]
HMIT	*SLC2A 13*	Chr.5	10	NC_037332.1	NP_001179892	648 aa	Brain	H^+^/myo-inositol transporter	[86]

**Table 3:** mRNA expression of *SLC2A* genes in different animals and tissue under heat stress.

Protein	Gene	Animals	Tissue	mRNA Expression	Reference
		Buffalos	Blood	Up-regulated	[23]
GLUT1	*SLC2A1*	Chicken	Intestine	Down-regulated	[22]
		Chicken	Intestine	Down-regulated	[89]
GLUT2	*SLC2A2*	Pigs	Intestine	Up-regulated	[91]
GLUT3	*SLC2A3*	Boar	Sertoli cells	Down-regulated	[93]
GLUT4	*SLC2A4*	Pigs	Liver, Muscle	Up-regulated	[92]
GLUT5	*SLC2A5*	Chicken	Intestine	Up-regulated	[22]
GLUT10	*SLC2A10*	Chicken	Intestine	Up-regulated	[22]

## References

[1] Cankaya M., Hernandez A.M., Ciftci M., Beydemir S., Ozdemir H., Budak H., Gulcin I., Comakli V., Emircupani T., Ekinci D. (2007). An analysis of expression patterns of genes encoding proteins with catalytic activities. BMC Genom..

[2] Nozière P., Ortigues-Marty I., Loncke C., Sauvant D. (2010). Carbohydrate quantitative digestion and absorption in ruminants: From feed starch and fibre to nutrients available for tissues. Animal.

[3] Mayes P.A. (1996). Gluconeogenesis and control of the blood glucose. Harper’s Biochemistry.

[4] Liu H., Zhao K., Liu J. (2013). Effects of Glucose Availability on Expression of the Key Genes Involved in Synthesis of Milk Fat, Lactose and Glucose Metabolism in Bovine Mammary Epithelial Cells. PLoS ONE.

[5] Scroll P., For D. (2007). Potential Function of Its Novel Members. Mol. Membr. Biol..

[6] Zhao F.Q. (2014). Biology of glucose transport in the mammary gland. J. Mammary Gland. Biol. Neoplasia.

[7] Braun E.J., Sweazea K.L. (2008). Glucose regulation in birds. Comp. Biochem. Physiol. B Biochem. Mol. Biol..

[8] Thorens B. (1996). Glucose transporters in the regulation of intestinal, renal, and liver glucose fluxes. Am. J. Physiol. Gastrointest. Liver Physiol..

[9] Das R., Sailo L., Verma N., Bharti P., Saikia J., Imtiwati, Kumar R. (2016). Impact of heat stress on health and performance of dairy animals: A review. Vet. World.

[10] Collier R.J., Baumgard L.H., Zimbelman R.B., Xiao Y. (2019). Heat stress: Physiology of acclimation and adaptation. Anim. Front..

[11] Sammad A., Wang Y.J., Umer S., Lirong H., Khan I., Khan A., Ahmad B., Wang Y. (2020). Nutritional physiology and biochemistry of dairy cattle under the influence of heat stress: Consequences and opportunities. Animals.

[12] Koubková M., Knížková I., Kunc P., Härtlová H., Flusser J., Doležal O. (2002). Influence of high environmental temperatures and evaporative cooling on some physiological, hematological and biochemical parameters in high-yielding dairy cows. Czech J. Anim. Sci..

[13] Moseley P. (2000). Stress proteins and the immune response. Immunopharmacology.

[14] Gupta M., Kumar S., Dangi S., Jangir B. (2015). Physiological, Biochemical and Molecular Responses to Thermal Stress in Goats. Int. J. Livest. Res..

[15] Dervisevik M., Dinevska-Kjovkarovska S., Miova B., Mitev S., Velkovski M., Susleski D. (2011). Heat acclimation-induced changes in heart glycogen/glucose metabolism in rats. J. Physiol. Sci..

[16] Garriga C., Hunter R.R., Amat C., Planas J.M., Mitchell M.A., Moretó M. (2005). Heat stress increases apical glucose transport in the chicken jejunum. Am. J. Physiol. Regul. Integr. Comp. Physiol..

[17] Horowitz M. (2003). Matching the Heart to Heat-Induced Circulatory Load: Heat-Acclimatory Responses. News Physiol. Sci..

[18] Rhoads M.L., Rhoads R.P., VanBaale M.J., Collier R.J., Sanders S.R., Weber W.J., Crooker B.A., Baumgard L.H. (2009). Effects of heat stress and plane of nutrition on lactating Holstein cows: I. Production, metabolism, and aspects of circulating somatotropin. J. Dairy Sci.

[19] Wheelock J.B., Rhoads R.P., VanBaale M.J., Sanders S.R., Baumgard L.H. (2010). Effects of heat stress on energetic metabolism in lactating Holstein cows. J. Dairy Sci..

[20] Baumgard L.H., Wheelock J.B., Sanders S.R., Moore C.E., Green H.B., Waldron M.R., Rhoads R.P. (2011). Postabsorptive carbohydrate adaptations to heat stress and monensin supplementation in lactating Holstein cows. J. Dairy Sci..

[21] Sano H., Ambo K., Tsuda T. (1985). Blood Glucose Kinetics in Whole Body and Mammary Gland of Lactating Goats Exposed to Heat. J. Dairy Sci..

[22] Habashy W.S., Milfort M.C., Fuller A.L., Attia Y.A., Rekaya R., Aggrey S.E. (2017). Effect of heat stress on protein utilization and nutrient transporters in meat-type chickens. Int. J. Biometeorol..

[23] Singh R., Rajesh C., Mishra S.K., Gurao A., Vohra V., Niranjan S.K., Kataria R.S. (2018). Comparative Expression Profiling of Heat-stress Tolerance Associated HSP60 and GLUT-1 Genes in Indian Buffaloes. Indian J. Dairy Sci..

[24] Donkin S.S., Hammon H. (2005). Chapter 15 Hepatic gluconeogenesis in developing ruminants. Biology of Growing Animals.

[25] Ocquettea J.H., Beb H.A. (2000). Facilitative glucose transporters in livestock species. Reprod. Nutr. Dev..

[26] Reynolds C.K. Glucose Balance in Cattle. Proceedings of the Florida Ruminant Nutrition Symposium.

[27] Threadgold L.C., Kuhn N.J. (1979). Glucose-6-phosphate hydrolysis by lactating rat mammary gland. Int. J. Biochem..

[28] Kronfeld D.S. (1982). Major Metabolic Determinants of Milk Volume, Mammary Efficiency, and Spontaneous Ketosis in Dairy Cows. J. Dairy Sci..

[29] Bell A.W. (1995). Regulation of organic nutrient metabolism during transition from late pregnancy to early lactation. J. Anim. Sci..

[30] Drackley J., Overton T., Douglas G. (2001). Adaptations of Glucose and Long-Chain Fatty Acid Metabolism in Liver of Dairy Cows during the Periparturient Period. J. Dairy Sci..

[31] Nayeri S., Stothard P. (2016). Tissues, Metabolic Pathways and Genes of Key Importance in Lactating Dairy Cattle. Springer Sci. Rev..

[32] Harper D., Chandler B. (2016). Splanchnic circulation. BJA Educ..

[33] Bickerstaffe R., Annison E.F., Linzell J.L. (1974). The metabolism of glucose, acetate, lipids and amino acids in lactating dairy cows. J. Agric. Sci..

[34] Lin Y., Sun X., Hou X., Qu B., Gao X., Li Q. (2016). Effects of glucose on lactose synthesis in mammary epithelial cells from dairy cow. BMC Vet. Res..

[35] Rhoads R.P., Nardone A., Ronchi B., Bernabucci U., Lacetera N., Baumgard L.H. (2010). Metabolic and hormonal acclimation to heat stress in domesticated ruminants. Animal.

[36] Guo J., Gao S., Quan S., Zhang Y., Bu D., Wang J. (2018). Blood amino acids profile responding to heat stress in dairy cows. Asian Australas. J. Anim. Sci..

[37] Lin H., Du R., Gu X.H., Li F.C., Zhang Z.Y. (2000). A study on the plasma biochemical indices of heat-stressed broilers. Asian Australas. J. Anim. Sci..

[38] Miova B., Dinevska-Kjovkarovska S., Cvetkovska F., Mitev S., Dzimrevska A., Dimitrovska M. (2013). Liver carbohydrate metabolism in rats in the period of recovery after acute heat stress. Maced. J. Med Sci..

[39] O’Brien M.D., Rhoads R.P., Sanders S.R., Duff G.C., Baumgard L.H. (2010). Metabolic adaptations to heat stress in growing cattle. Domest. Anim. Endocrinol..

[40] Settivari R., Spain J., Ellersieck M., Byatt J., Collier R., Spiers D. (2007). Relationship of Thermal Status to Productivity in Heat-Stressed Dairy Cows Given Recombinant Bovine Somatotropin. J. Dairy Sci..

[41] Baumgard L.H., Rhoads R.P. (2013). Effects of Heat Stress on Postabsorptive Metabolism and Energetics. Annu. Rev. Anim. Biosci..

[42] Drackley J.K. (1999). ADSA foundation scholar award: Biology of dairy cows during the transition period: The final frontier?. J. Dairy Sci..

[43] Koch F., Lamp O., Eslamizad M., Weitzel J., Kuhla B. (2016). Metabolic Response to heat stress in late-pregnant and early lactation dairy cows: Implications to liver-muscle crosstalk. PLoS ONE.

[44] Kvidera S.K., Horst E.A., Abuajamieh M., Mayorga E.J., Fernandez M.V.S., Baumgard L.H. (2017). Glucose requirements of an activated immune system in lactating Holstein cows. J. Dairy Sci..

[45] Alamer M. (2011). The role of prolactin in thermoregulation and water balance during heat stress in domestic ruminants. Asian J. Anim. Vet. Adv..

[46] Ahmed N., Berridge M.V. (1998). Transforming oncogenes regulate glucose transport by increasing transporter affinity for glucose: Contrasting effects of oncogenes and heat stress in a murine marrow-derived cell line. Life Sci..

[47] Knapp D.M., Grummer R.R. (1991). Response of Lactating Dairy Cows to Fat Supplementation During Heat Stress. J. Dairy Sci..

[48] Sammad A., Umer S., Shi R., Zhu H., Zhao X., Wang Y. (2020). Dairy cow reproduction under the influence of heat stress. J. Anim. Physiol. Anim. Nutr..

[49] Nafikov R.A., Beitz D.C. (2007). Carbohydrate and Lipid Metabolism in Farm Animals. J. Nutr..

[50] James A., DeShazer James A. (2013). DeShazer Livestock Energetics and Thermal Environmental Management. Livest. Energetics Therm. Environ. Manag..

[51] Baumgard L.H., Wheelock J.B., Shwartz G., O’Brien M., VanBaale M.J., Collier R.J., Rhoads M.L., Rhoads R.P. Effects of Heat Stress on Nutritional Requirements of Lactating Dairy Cattle. Proceedings of the 5th Annual Arizona Dairy Production Conference.

[52] Cai L., Yu J., Hartanto R., Zhang J., Yang A., Qi D. (2019). Effects of heat challenge on growth performance, ruminal, blood and physiological parameters of Chinese crossbred goats. Small Rumin. Res..

[53] Fan C., Su D., Tian H., Li X., Li Y., Ran L., Hu R., Cheng J. (2018). Liver metabolic perturbations of heat-stressed lactating dairy cows. Asian Australas. J. Anim. Sci..

[54] McGuire M.A., Beede D.K., Collier R.J., Buonomo F.C., De Lorenzo M.A., Wilcox C.J., Huntington G.B., Reynolds C.K. (1991). Effects of acute thermal stress and amount of feed intake on concentrations of somatotropin, insulin-like growth factor (IGF)-I and IGF-II, and thyroid hormones in plasma of lactating Holstein cows. J. Anim. Sci..

[55] Yousef M.K., Johnson H.D. (1966). Calorigenesis of cattle as influenced by growth hormone and environmental temperature. J. Anim. Sci..

[56] Marinković M.D., Belić B., Cincović M.R., Đoković R., Lakić I., Stojanac N., Stevančević O., Devečerski G. (2019). Relationship between insulin, glucose, non-esterified fatty acid and indices of insuliresistance in obese cows during the dry period and early lactation. Acta Vet. Brno.

[57] Bauman D. (1993). Effects of Exogenous Bovine Somatotropin on Lactation. Annu. Rev. Nutr..

[58] Galster A.D., Clutter W.E., Cryer P.E., Collins J.A., Bier D.M. (1981). Epinephrine plasma thresholds for lipolytic effects in man. Measurements of fatty acid transport with [1-13C]palmitic acid. J. Clin. Investig..

[59] Streffer C. (1988). Aspects of Metabolic Change After Hyperthermia. Application of Hyperthermia in the Treatment of Cancer.

[60] Patel M.S., Korotchkina L.G. (2006). Regulation of the pyruvate dehydrogenase complex. Biochem. Soc. Trans..

[61] Rhoads R., Baumgard L.H., Suagee J.K. (2013). 2011 AND 2012 EARLY CAREERS ACHIEVEMENT AWARDS: Metabolic priorities during heat stress with an emphasis on skeletal muscle1,2. J. Anim. Sci..

[62] Rhoads R.P., La Noce A.J., Wheelock J.B., Baumgard L.H. (2011). Short communication: Alterations in expression of gluconeogenic genes during heat stress and exogenous bovine somatotropin administration. J. Dairy Sci..

[63] Tian H., Wang W., Zheng N., Cheng J., Li S., Zhang Y., Wang J. (2015). Identification of diagnostic biomarkers and metabolic pathway shifts of heat-stressed lactating dairy cows. J. Proteom..

[64] Bell A.W., Burhans W.S., Overton T.R. (2000). Protein nutrition in late pregnancy, maternal protein reserves and lactation performance in dairy cows. Proc. Nutr. Soc..

[65] Sejian V., Indu S., Naqvi S.M.K. (2013). Impact of short term exposure to different environmental temperature on the blood biochemical and endocrine responses of Malpura ewes under semi-arid tropical environment. Indian J. Anim. Sci..

[66] Gonzalez-Rivas P.A., Chauhan S.S., Ha M., Fegan N., Dunshea F.R., Warner R.D. (2020). Effects of heat stress on animal physiology, metabolism, and meat quality: A review. Meat Sci..

[67] Schneider P.L., Beede D.K., Wilcox C.J. (1988). Nycterohemeral patterns of acid-base status, mineral concentrations and digestive function of lactating cows in natural or chamber heat stress environments. J. Anim. Sci..

[68] Joksimovic-Todorovic M., Davidovic V., Hristov S., Stankovic B. (2011). Effect of heat stress on milk production in dairy cows. Biotechnol. Anim. Husb..

[69] Ikari A., Nakano M., Suketa Y., Harada H., Takagi K. (2005). Reorganization of ZO-1 by sodium-dependent glucose transporter activation after heat stress in LLC-PK1 cells. J. Cell. Physiol..

[70] Febbraio M.A. (2001). Alterations in energy metabolism during exercise and heat stress. Sports Med..

[71] Mehaba N., Salama A.A., Such X., Albanell E., Caja G. (2019). Lactational Responses of Heat-Stressed Dairy Goats to Dietary L-Carnitine Supplementation. Animals.

[72] Mueckler M. (1994). Facilitative glucose transporters. Eur. J. Biochem..

[73] Ostrowska M., Jarczak J., Zwierzchowski L. (2015). Glucose transporters in cattle—A review. Anim. Sci. Pap. Rep..

[74] Wood I.S., Trayhurn P. (2003). Glucose transporters (GLUT and SGLT): Expanded families of sugar transport proteins. Br. J. Nutr..

[75] Uldry M., Thorens B. (2004). The SLC2 family of facilitated hexose and polyol transporters. Pflug. Arch. Eur. J. Physiol..

[76] Zhao F.Q., Dixon W.T., Kennelly J.J. (1996). Localization and gene expression of glucose transporters in bovine mammary gland. Comp. Biochem. Physiol. B Biochem. Mol. Biol..

[77] Zhao F.Q., Okine E.K., Kennelly J.J. (1999). Glucose transporter gene expression in bovine mammary gland. J. Anim. Sci..

[78] Zhao F.Q., Glimm D.R., Kennelly J.J. (1993). Distribution of mammalian facilitative glucose transporter messenger rna in bovine tissues. Int. J. Biochem..

[79] Liao S.F., Harmon D.L., Vanzant E.S., McLeod K.R., Boling J.A., Matthews J.C. (2010). The small intestinal epithelia of beef steers differentially express sugar transporter messenger ribonucleic acid in response to abomasal versus ruminal infusion of starch hydrolysate. J. Anim. Sci..

[80] Nishimoto H., Matsutani R., Yamamoto S., Takahashi T., Hayashi K.G., Miyamoto A., Hamano S., Tetsuka M. (2006). Gene expression of glucose transporter (GLUT) 1, 3 and 4 in bovine follicle and corpus luteum. J. Endocrinol..

[81] Augustin R., Pocar P., Navarrete-Santos A., Wrenzycki C., Gandolfi F., Niemann H., Fischer B. (2001). Glucose transporter expression is developmentally regulated in in vitro derived bovine preimplantation embryos. Mol. Reprod. Dev..

[82] Zhao F.Q., Miller P.J., Wall E.H., Zheng Y.C., Dong B., Neville M.C., McFadden T.B. (2004). Bovine glucose transporter GLUT8: Cloning, expression, and developmental regulation in mammary gland. Biochim. Biophys. Acta Gene Struct. Expr..

[83] McVie-Wylie A.J., Lamson D.R., Chen Y.T. (2001). Molecular cloning of a novel member of the GLUT family of transporters, SLC2A10 (GLUT10), localized on chromosome 20q13.1: A candidate gene for NIDDM susceptibility. Genomics.

[84] Wu X., Li W., Sharma V., Godzik A., Freeze H.H. (2002). Cloning and characterization of glucose transporter 11, a novel sugar transporter that is alternatively spliced in various tissues. Mol. Genet. Metab..

[85] Komatsu T., Itoh F., Sakumoto R., Hodate K., Obara Y., Kushibiki S. (2007). Changes in the gene expression of adiponectin and glucose transporter 12 (GLUT12) in lactating and non-lactating cows. Anim. Sci. J..

[86] Uldry M., Ibberson M., Horisberger J.D., Chatton J.Y., Riederer B.M., Thorens B. (2001). Identification of a mammalian H+-myo-inositol symporter expressed predominantly in the brain. EMBO J..

[87] Malago J.J., Koninkx J.F.J.G., Van Dijk J.E. (2002). The heat shock response and cytoprotection of the intestinal epithelium. Cell Stress Chaperones.

[88] Simpson I.A., Appel N.M., Hokari M., Oki J., Holman G.D., Maher F., Koehler-Stec E.M., Vannucci S.J., Smith Q.R. (1999). Blood-brain barrier glucose transporter: Effects of hypo and hyperglycemia revisited. J. Neurochem..

[89] Sun X., Zhang H., Sheikhahmadi A., Wang Y., Jiao H., Lin H., Song Z. (2014). Effects of heat stress on the gene expression of nutrient transporters in the jejunum of broiler chickens (Gallus gallus domesticus). Int. J. Biometeorol..

[90] Bánhegyi G., Benedetti A., Margittai É., Marcolongo P., Fulceri R., Németh C.E., Szarka A. (2014). Subcellular compartmentation of ascorbate and its variation in disease states. Biochim. Biophys. Acta Mol. Cell Res..

[91] Pearce S.C., Mani V., Boddicker R.L., Johnson J.S., Weber T.E., Ross J.W., Rhoads R.P., Baumgard L.H., Gabler N.K. (2013). Heat Stress Reduces Intestinal Barrier Integrity and Favors Intestinal Glucose Transport in Growing Pigs. PLoS ONE.

[92] Cervantes M., Cota M., Arce N., Castillo G., Avelar E., Espinoza S., Morales A. (2016). Effect of heat stress on performance and expression of selected amino acid and glucose transporters, HSP90, leptin and ghrelin in growing pigs. J. Therm. Biol..

[93] Bao Z.Q., Liao T.T., Yang W.R., Wang Y., Luo H.Y., Wang X.Z. (2017). Heat stress–induced autophagy promotes lactate secretion in cultured immature boar Sertoli cells by inhibiting apoptosis and driving SLC2A3, LDHA, and SLC16A1 expression. Theriogenology.

[94] Gaughan J., Lacetera N., Valtorta S.E., Khalifa H.H., Hahn L., Mader T. (2009). Response of Domestic Animals to Climate Challenges. Biometeorology for Adaptation to Climate Variability and Change. Biometeorology.

[95] Horowitz M. (2002). From molecular and cellular to integrative heat defense during exposure to chronic heat. Comp. Biochem. Physiol. A Mol. Integr. Physiol..

[96] Hall D.M., Buettner G.R., Oberley L.W., Xu L., Matthes R.D., Gisolfi C.V. (2001). Mechanisms of circulatory and intestinal barrier dysfunction during whole body hyperthermia. Am. J. Physiol. Heart Circ. Physiol..

[97] Hall D.M., Baumgardner K.R., Oberley T.D., Gisolfi C.V. (1999). Splanchnic tissues undergo hypoxic stress during whole body hyperthermia. Am. J. Physiol. Gastrointest. Liver Physiol..

[98] Jing L., Zhang R., Liu Y., Zhu W., Mao S. (2014). Intravenous lipopolysaccharide challenge alters ruminal bacterial microbiota and disrupts ruminal metabolism in dairy cattle. Br. J. Nutr..

[99] Lambert G.P., Gisolfi C.V., Berg D.J., Moseley P.L., Oberley L.W., Kregel K.C. (2002). Selected contribution: Hyperthermia-induced intestinal permeability and the role of oxidative and nitrosative stress. J. Appl. Physiol..

[100] Leon L.R. (2007). Heat stroke and cytokines. Prog. Brain Res..

[101] Krajmalnik-Brown R., Ilhan Z.E., Kang D.W., DiBaise J.K. (2012). Effects of gut microbes on nutrient absorption and energy regulation. Nutr. Clin. Pract..

[102] Carabaño M.J., Ramón M., Menéndez-Buxadera A., Molina A., Díaz C. (2019). Selecting for heat tolerance. Anim. Front..

[103] Xu Q., Wang Y.C., Hu L.R., Kang L. The effect of temperature stress on milk production traits and blood biochemical parameters of Chinese Holstein cows. Proceedings of the World Congress on Genetics Applied to Livestock Production.

[104] West J.W., Mullinix B.G., Bernard J.K. (2003). Effects of hot, humid weather on milk temperature, dry matter intake, and milk yield of lactating dairy cows. J. Dairy Sci..

[105] Amamou H., Beckers Y., Mahouachi M., Hammami H. (2019). Thermotolerance indicators related to production and physiological responses to heat stress of holstein cows. J. Therm. Biol..

[106] Ng D.P.K., Canani L., Araki S.I., Smiles A., Moczulski D., Warram J.H., Krolewski A.S. (2002). Minor effect of GLUT1 polymorphisms on susceptibility to diabetic nephropathy in type 1 diabetes. Diabetes.

[107] Grabellus F., Sheu S.Y., Bachmann H.S., Lehmann N., Otterbach F., Heusner T.A., Antoch G., Bockisch A., Kimmig R., Schmid K.W. (2010). The XbaI G>T polymorphism of the glucose transporter 1 gene modulates 18F-FDG uptake and tumor aggressiveness in breast cancer. J. Nucl. Med..

[108] Seefried F.R. (2008). Genomic Characterisation and Polymorphism Analysis of Candidate Genes for Milk Production Traits and Association Studies in Three Cattle Breeds. Ph.D. Thesis.

[109] Herbut P., Angrecka S., Godyń D. (2018). Effect of the duration of high air temperature on cow’s milking performance in moderate climate conditions. Ann. Anim. Sci..

[110] Berman A. (2019). An overview of heat stress relief with global warming in perspective. Int. J. Biometeorol..

[111] Turner L.W., Chastain J.P., Hemken R.W., Gates R.S., Crist W.L. (1992). Reducing Heat Stress in Dairy Cows Through Sprinkler and Fan Cooling. Appl. Eng. Agric..

[112] Kadzere C.T., Murphy M.R., Silanikove N., Maltz E. (2002). Heat stress in lactating dairy cows: A review. Livest. Prod. Sci..

[113] Lees A.M., Sejian V., Wallage A.L., Steel C.C., Mader T.L., Lees J.C., Gaughan J.B. (2019). The impact of heat load on cattle. Animals.

[114] Liu J., Li L., Chen X., Lu Y., Wang D. (2019). Effects of heat stress on body temperature, milk production, and reproduction in dairy cows: A novel idea for monitoring and evaluation of heat stress—A review. Asian Australas. J. Anim. Sci..

[115] Tang Y., Li J., Liao S., Qi M., Kong X., Tan B., Yin Y., Wang J. (2018). The effect of dietary protein intake on immune status in pigs of different genotypes. Food Agric. Immunol..

[116] Caroprese M., Albenzio M., Marino R., Santillo A., Sevi A. (2013). Dietary glutamine enhances immune responses of dairy cows under high ambient temperature. J. Dairy Sci..

[117] Wu G., Meier S.A., Knabe D.A. (1996). Dietary Glutamine Supplementation Prevents Jejunal Atrophy in Weaned Pigs. J. Nutr..

[118] Sciences D. (2014). Effect of trace mineral supplementation on selected minerals, energy metabolites, oxidative stress and immune parameters and its association with uterine diseases in dairy cattle. J. Dairy Sci..

[119] Yadav B., Singh G., Wankar A., Dutta N., Chaturvedi V.B., Verma M.R. (2016). Effect of simulated heat stress on digestibility, methane emission and metabolic adaptability in crossbred cattle. Asian Australas. J. Anim. Sci..

[120] West J.W. (1999). Nutritional strategies for managing the heat-stressed dairy cow. J. Anim. Sci..

[121] Zhang Z., La S.K., Zhang G.W., Du H.S., Wu Z.Z., Wang C., Liu Q., Guo G., Huo W.J., Zhang J. (2020). Diet supplementation of palm fat powder and coated folic acid on performance, energy balance, nutrient digestion, ruminal fermentation and blood metabolites of early lactation dairy cows. Anim. Feed Sci. Technol..

[122] Palmquist D.L., Jenkins T.C. (1980). Fat in Lactation Rations: Review. J. Dairy Sci..

[123] Arieli A., Adin G., Bruckental I. (2004). The effect of protein intake on performance of cows in hot environmental temperatures. J. Dairy Sci..

[124] Bruno R.G.S., Rutigliano H.M., Cerri R.L., Robinson P.H., Santos J.E.P. (2009). Effect of feeding Saccharomyces Cerevisiae on performance of dairy cows during summer heat stress. Anim. Feed Sci. Technol..

[125] Wang B., Wang C., Guan R., Shi K., Wei Z., Liu J., Liu H. (2019). Effects of dietary rumen-protected betaine supplementation on performance of postpartum dairy cows and immunity of newborn calves. Animals.

[126] Erdman R.A. (1988). Dietary Buffering Requirements of the Lactating Dairy Cow: A Review. J. Dairy Sci..

[127] Melendez P., Donovan G.A., Risco C.A., Littell R., Goff J.P. (2003). Effect of calcium-energy supplements on calving-related disorders, fertility and milk yield during the transition period in cows fed anionic diets. Theriogenology.

[128] Costanzo A.D.I., Spiers D.E. (1997). Supplementation of Nicotinic Acid for Lactating Holstein Cows Under Heat Stress Conditions. J. Dairy Sci..

[129] Barreras A., Castro-Pérez B.I., López-Soto M.A., Torrentera N.G., Montaño M.F., Estrada-Angulo A., Ríos F.G., Dávila-Ramos H., Plascencia A., Zinn R.A. (2013). Influence of ionophore supplementation on growth performance, dietary energetics and carcass characteristics in finishing cattle during period of heat stress. Asian Australas. J. Anim. Sci..

[130] Li G., Chen S., Chen J., Peng D., Gu X. (2020). Predicting rectal temperature and respiration rate responses in lactating dairy cows exposed to heat stress. J. Dairy Sci..

[131] Yano M., Shimadzu H., Endo T. (2014). Modelling temperature effects on milk production: A study on Holstein cows at a Japanese farm. Springer Plus.

[132] Nguyen T.T.T., Bowman P.J., Haile-Mariam M., Nieuwhof G.J., Hayes B.J., Pryce J.E. (2017). Short communication: Implementation of a breeding value for heat tolerance in Australian dairy cattle. J. Dairy Sci..

[133] Berman A. (2011). Invited review: Are adaptations present to support dairy cattle productivity in warm climates?. J. Dairy Sci..

